# Understanding street protests: from a mathematical model to protest management

**DOI:** 10.1371/journal.pone.0319837

**Published:** 2025-04-10

**Authors:** Sergei Petrovskii, Maxim Shishlenin, Anton Glukhov

**Affiliations:** 1 School of Computing and Mathematical Sciences, University of Leicester, Leicester, UK; 2 Peoples Friendship University of Russia (RUDN University), 6 Miklukho-Maklaya Str., 117198 Moscow, Russia; 3 Sobolev Institute of Mathematics, Novosibirsk, Russia; University of Exeter, UNITED KINGDOM OF GREATBRITAIN AND NORTHERN IRELAND

## Abstract

Street protests have been a common feature of human society for many centuries. They often act as a driver of social changes but they may also disrupt everyday life and lead to considerable economic losses. Understanding of factors that may affect the duration of street protests and the number of participants is a problem of pivotal importance. Mathematical modelling is an efficient research approach to study this problem. Here we present a novel modelling framework that takes into account heterogeneity of protesters behaviour and the effect of policing. Using the 2018–2019 Yellow Vest Movement in France as a case study, we show that our model is in a very good agreement with data. We also show that a moderate increase in the efficiency of police actions on particular days may have a significant effect on protest’s intensity and duration. Our findings open a possibility for a more efficient protests management.

## Introduction

Social protest––understood here as a public expression of objection or disapproval towards an idea or action, typically of a political or economic origin––has been a ubiquitous feature of human society for many centuries. It is an important driver of social dynamics and, ultimately, social progress. Social protests may take a variety of different forms as well as differ hugely in their duration and the number of participants. It can range from an occasional disapproval voiced out by a small group of people, perhaps with little or no effect, to repeated massive street protests, demonstrations and even riots that can shake the corresponding society and lead to landslide changes in the governance and social system. Some recent examples are given by London 2011 riots, 2010–2012 Arab Spring, 2018–2019 Yellow Vests Movement in France, 2019 riots in Santiago, and 2019–2020 Hong Kong riots, to name just a few.

Whatever is the goal of a particular social protest, the likeliness of success apparently depends on the protest intensity (i.e., the number of active participants) and duration. While a single event of public disapproval of, for instance, an unpopular government decision, even if attended by many people, is unlikely to result in revoking it, a series of repeated similar events is much more likely to achieve the goal. Understanding factors that can affect protest’s intensity and duration is therefore not only a problem of considerable theoretical interest but also of high practical importance.

Social protest is a complex phenomenon and a variety of scientific approaches have been developed to study it. Traditional approaches usually employ concepts, methods and tools of social sciences and psychology [1–4]. That has often been combined with or supported by statistical analysis of data, e.g., estimating the number of street protesters [5–8] and sometimes their location and/or spatial distribution [9–11], analysing their social identification [12,13], revealing the number of police involved [14], etc.

Insightful as they are, methods of social sciences are not always powerful enough when a more quantitative analysis is needed. Complementary or alternative research approaches are based on mathematical models of human behaviour [15] and complex systems science [16,17], which in turn involve a broad range of methods and utilizes a variety of specific modelling techniques [18–28]. In particular, the ‘epidemiological’ approach originally suggested by Epstein [29] has been shown to be an efficient modelling framework to describe street protests [30–33]. According to this approach, the population is structured into several groups, e.g. the active street protesters (that can be formally regarded as ‘infected’ with a certain kind of behaviour or opinion), the former protesters that retired from the movement, and the rest of the adult population regarded as ‘susceptible’ because they can incur the opinion to join the protest. The arising mathematical models are close to those that are commonly used in epidemics modelling [34]. We mention here that mathematically similar models of opinion dynamics have been successfully applied to a number of problems of various origin, including social phenomena as diverse as smoking [35], fashion [36] and elections [37].

The paper is organized as follows. In the next section, we introduce our mathematical model that takes into account two different types of participants behavioural response (where one of the types accounts for collective effects) as well as the effect of policing. We then apply the model to a ‘case study’: the Yellow Vest Movement (YVM) in 2018–2019 in France. We first demonstrate that the model prediction is in good agreement with the data and show how model parameters can be restored from the data. We then focus on the effect of policing to reveal that its ‘efficiency’ (quantified as either the number of police force involved or the number of detained protesters per policeman) can be a crucial factor, so that a change in the police presence just by 10–20% may result in a disproportionate large change (more than 100%) in the protests intensity and/or duration.

## Mathematical model

**Fig 1:**
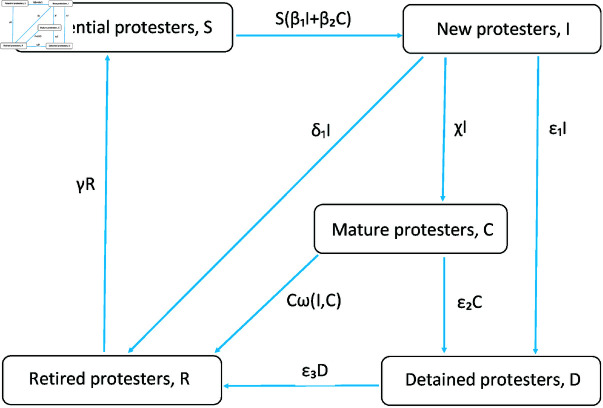
Flow chart diagram. Flow chart diagram describing the model structure. The boxes represent the groups into which the population is divided, the arrow show transitions between the groups. The arrows’ labels show the transition rates; see details in the text.

We use a compartment-type model where the population is divided into several groups according to people’s attitude (‘opinion’) towards an ongoing protest and/or their relevant experience, see Fig 1. Potential or ‘susceptible’ protesters (*S*) are people who do not currently participate in the protest but can join it if/when they develop a sufficiently strong supportive attitude. Protesters are divided into novice protesters (*I*) who have only recently joined the movement, and experienced (mature) protesters (*C*), i.e., people with a considerable previous experience in street protests. Arguably, such division is necessary, because the protesters are likely to behave differently depending on the extent of their personal experience (see below). We mention here that in several countries in the world, in particular in France - recall that we are going to focus on the *gilets jaunes* movement in France as our main case study - there is a distinct ‘culture of protests’ [38] so that its history includes more than a dozen of public events of protest (sometimes involving violence) already in the first two decades of the 21st century [39]. It is therefore plausible to assume that a certain fraction of protesters may have some experience in participating in similar events.

Furthermore, protesters can retire (withdraw) from the protests for a variety of reasons, e.g., becoming disillusioned with the goals of the movement or because of some personal or family issues. We assume that those reasons have an effect lasting long enough, so that the retired protesters do not re-enter the susceptible group immediately but form their own one (*R*). Finally, we assume that the protest is dealt with by the police, so that a certain number of protesters is detained (*D*); this quantity can be regarded as a proxy for the efficiency of police actions. We assume that the experience of being imprisoned is, on average, stressful enough to prevent the detained participants to immediately re-join, after their release, the susceptible group; instead, they join the retired group. The number of people in each group is assumed to change continuously with time due to the transitions between the groups (see Fig 1).

The key point of our model is that ‘susceptible’ individuals can become ‘infected’ with the opinion to join a street protest, hence changing group *S* to group *I* with a certain transition rate. We assume that this can happen when they are exposed to the ideas of the movement, e.g., either as a result of face-to-face contacts with individuals from groups *I* or *C* who are already actively participating in the protests or through corresponding contacts via social networks. Correspondingly, the transition rate is proportional to the frequency of such contacts and hence is determined by the mass action law. (Mass action law in epidemiology states that the rate of increase in epidemic incidence is proportional to the product of the number of infectious and susceptible individuals, e.g., see [34].) Novice protesters can retire (transition *I* → *R*) or stay and eventually become mature ones (*I* → *C*). Also, retired protesters can eventually become susceptible again (*R* → *S*). We assume that, in all three cases, the decisions are made individually, hence neglecting any collective effects.

The corresponding transition rates are then linear functions of, respectively, variables *I* and *R* (see Fig 1). Similarly, we assume that the rate at which protesters are detained is proportionate to the frequency of contacts between the protesters and the police (see details below) and is described by the mass action law too.

Mathematically, the conceptual model shown in Fig 1 is described by the following equations:


$$\begin{array}{ccc} \frac{dS}{dt} & =-\beta_{1}SI-\beta_{2}SC+\gamma R, & \end{array}$$ (1)

$$\begin{array}{ccc} \frac{dI}{dt} & =\beta_{1}SI+\beta_{2}SC-\chi I-\delta_{1}I-\varepsilon_{1}I, & \end{array}$$ (2)

$$\begin{array}{ccc} \frac{dC}{dt} & =\chi I-C\omega (I,C)-\varepsilon_{2}C, & \end{array}$$ (3)

$$\begin{array}{ccc} \frac{dR}{dt} & =\delta_{1}I+C\omega (I,C)-\gamma R+\varepsilon_{3}D, & \end{array}$$ (4)

$$\begin{array}{ccc} \frac{dD}{dt} & =\varepsilon_{1}I+\varepsilon_{2}C-\varepsilon_{3}D, & \end{array}$$ (5)

where *t* is time and *ω* ( *I* , *C* )  is the withdrawal rate of mature (experienced) protesters.

We further take into account that, while the linear retirement rate is likely to be true for novice protesters, it may not necessarily be true for experienced (mature) ones because of changes in their behaviour. Since experienced protesters have already spent some considerable time participating together in street actions, it seems likely that they have established strong ties between themselves, in particular based on shared in group emotions [13,40]. It has indeed been observed that participants in a collective action learn from their experience and tend to adapt their behaviour making the collective effects stronger [41].

To account for the effect of collective behaviour, we assume that the probability of a mature protester to retire is a decreasing function of the total number of protesters *N* = *I* + *C*, so that the per capita retirement rate *ω* ( *N* )  reaches its largest value when the number of protesters is small. More specifically, we consider the following hypothetical parametrization:


$$\begin{array}{ccc} \omega (I+C)=\delta_{2}+(\delta_{1}-\delta_{2})\frac{C_{0}^{n}}{{\left(I+C\right)}^{n}+C_{0}^{n}}, & & \end{array}$$ (6)

where $\delta_{2},C_{0}$ and *n* are additional parameters. With an increase in *N*, the withdrawal rate of experienced participants *ω* monotonically decreases from $\delta_{1}$ to $\delta_{2}$. The difference $\delta_{0}=\delta_{1}-\delta_{2}$ therefore quantifies the strength of collective effects. $C_{0}$ describes the threshold number of protest participants when the collective effects become significant.

We mention that, according to the mass action law, the rates at which protesters are being detained by the police should also be a bilinear function of the corresponding variables, i.e., $\varepsilon_{1}^{\prime }FI$ and $\varepsilon_{2}^{\prime }FC$ for the novice and experienced protesters, respectively, where *F* is the size of the police force involved and $\varepsilon_{1,2}^{\prime }$ are the detention rates. In order to keep the model as simple as possible, here we assume that *F* is fixed. Having introduced new parameters as $\varepsilon_{1}=\varepsilon_{1}^{\prime }F$ and $\varepsilon_{2}=\varepsilon_{2}^{\prime }F$, we arrive at the corresponding terms in , (3) and (5).

Interpretation of group *S* in a more sociological or psychological context requires a discussion. An intuitive approach to this issue would probably lead to a definition that the susceptible group consists of strong supporters of the movement who are not yet active participants but can join the protests at any moment. However, such definition would lead to a high uncertainty in determining who actually belongs to group *S*, as support can have different grades and the difference between ‘strong’ and ‘weak’ is not always obvious. Moreover, the overall level of support can change considerably with time; for example, in the case of YVM it was varying between 50-70% of the general adult population [42]. In order to avoid this uncertainty and related arising technical difficulties, we use a weaker definition of potential protesters, i.e., these are people who, not presently being active participants, *can become ones if/when they develops* a sufficiently strong supportive attitude. Correspondingly, for the purposes of this study we assume that the susceptible group includes virtually all general population, perhaps only excluding children, old people, and possibly people with certain disabilities that cannot attend street protests for physical or legal reasons.

We mention here that, as an immediate consequence of the above, –5) form a closed system, so that the total population *P* remains approximately constant throughout the duration of the protests, *S* ( *t* ) + *I* ( *t* ) + *C* ( *t* ) + *R* ( *t* ) + *D* ( *t* ) = *P* ( *t* ) = const = *P* ( 0 ) . Thus, the model does not take into account changes in the population size due to demographic processes. This simplification is justified by the fact that street protests usually occur on a timescale much shorter than the typical timescale of population changes.

Note that our model contains a relatively large number of parameters (i.e., twelve in –6)). This can be regarded by some as a drawback of the model. Reference to a famous saying by von Neumann is often recalled on this occasion: “With four parameters I can fit an elephant, and with five I can make him wiggle his trunk” [43]. The point that is usually missed here is that von Neumann was referring to *arbitary* parameters. In contrast, the parameters in our model are by no means arbitrary. Indeed, they all have a clearly defined meaning and their value can be obtained from data, e.g. by considering the corresponding inverse problem [32]. Moreover, it seems to be obvious that, the more detailed is the model, the larger the number of parameters is; arguably, this trade-off is entirely unavoidable.

The goal of this paper is to demonstrate the capacity of the model given by –5) to describe the course of street protests, its power as a research tool to investigate various scenarios in protests dynamics, and to identify possible ways to control protests intensity and duration. That will be done by considering the case study below.

## Case study: the Yellow Vest Movement

### Data

**Fig 2:**
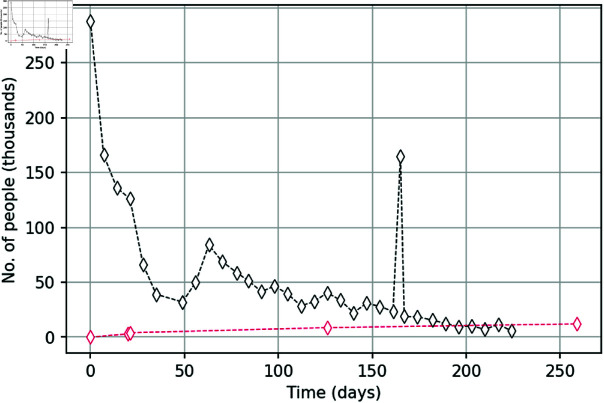
Data on the number of participants. Data on the number of active participants on a given date (black diamonds) and the total number of participants arrested by a given date (red diamonds) during the “Yellow Vests” social movement. Protests in much smaller numbers continued after week 32 but reliable data are not available.

The Yellow Vest Movement (YVM) in France took place during 2018-2019 as a response to the unpopular government decision to increase the fuel tax. In order to express their disapproval, on Saturday November 17, 2018, approximately 288,000 people took part in street protests across the country. The street actions then repeated every following Saturday for more than 30 weeks, albeit in smaller and gradually decreasing numbers. The YVM is well documented and data on the numbers of street protesters at different dates are freely available; see Fig 2. Some data on the number of detained protesters are available as well (cf. the red line in Fig 2). Relative numbers of novice and experienced protesters are not available.

We assume that the YVM street protesters consisted predominantly of people between the ages of 20 and 64. According to statistics for 2020, this age group in France was about 55.5 million people. We use this information as an aggregate initial condition, i.e., for *S* ( 0 ) + *I* ( 0 ) + *C* ( 0 ) + *R* ( 0 ) + *D* ( 0 ) = 55 , 500 , 000.

We additionally assume that, at the onset of the YVM, the number of experienced protesters was small. Since data on their actual number are not available, for the sake of simplicity we assume it to be zero. Thus, we arrive at the following data that can be used as the initial conditions for the model (1-5):


$$\begin{array}{ccc} S(0)=55212000,I(0)=288000,C(0)=R(0)=D(0)=0. & & \end{array}$$ (7)

Note that the solution of model (1-5) gives the number of detainees (i.e. people kept in custody) at any given time. However, these data are not available; what is available instead is the number of arrests. How the number of arrested participants can be transformed into the number of detainees is not a simple question, as along with new arrests some of the protesters that have been arrested earlier are eventually released. Any information on a typical time spent in custody is missing. Moreover, data on the number of arrests are available in a mixed form, i.e. date-specific as well as cumulative, that is

(i) between 1-126 days, 8700 protesters were
(ii) between 1-259 days, 12100 protesters were
(iii) on day 21, 1500 protesters were

These discrepancies need to be accounted for when fitting the model to the data (i.e. in formulating the inverse problem method); for technical details, see S1 Appendix.

### Simulation results

**Fig 3:**
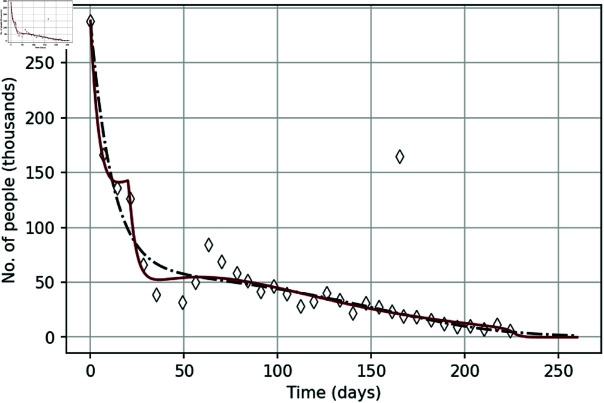
The solution of the model. Black dashed-and-dotted curve shows the number of street protesters *I* ( *t* ) + *C* ( *t* )  obtained as a solution of the ‘baseline’ model that does not account for detained participants (hence *D* ( *t* ) ≡ 0). To obtain the solution, parameter set q_∗_ was used, see S1 Table in S2 Appendix. Red solid curve shows the solution of the full model (1–5, 8) with the initial conditions (7) obtained for parameter set q (see S1 Table in S2 Appendix). Both parameter sets are revealed from the data using the inverse problem method, see S1 Appendix for details.

To begin with, we show how well –6) with the initial conditions (7) describes the course of the protests. The first step is to find the parameter values. This is done by applying the inverse problem approach (see S1 Appendix). Since we are particularly interested in revealing the effect of policing, we begin with the ‘baseline’ model (see Ref. [32]) that does not account for detained protesters, hence assuming that the police is either absent at all or chooses not to intervene. Correspondingly, *D* ( *t* ) ≡ 0, so that the full system (1–5) reduces to just –4) where $\varepsilon_{1}=\varepsilon_{2}=\varepsilon_{3}=0$. Applying the inverse problem approach, we obtain parameter values to which we refer as parameter set *q*_∗_; see S1 Table in S2 Appendix. Once the parameters are found, the second step is to solve the system (1-4) with the initial conditions (7). The results are shown in Fig 3 by the black dashed curve. Apparently, the general agreement between the model and the data is very good. However, there are some features of the data that the model does not reproduce, in particular the trough in weeks 5–8.

We now consider the full model (1–5) where we additionally take into account that the level of police presence and/or the efficiency of its actions is not uniform in time. In particular, the data on detained protesters indicates that police was handling the YVM protests on day 21 much harsher than on any other day. In terms of the model (1–5), it means that parameters $\varepsilon_{1}$ and $\varepsilon_{2}$ actually depend on time. Specifically, we consider the following piecewise-constant functions that combine an ‘active phase’ of the protests policing (larger $\varepsilon_{1}$ and $\varepsilon_{2}$) on day 21 with a ‘weak phase’ (smaller $\varepsilon_{1}$ and $\varepsilon_{2}$) during the rest of the protests:


$$\begin{array}{ccc} \varepsilon_{1}=\{\begin{array}{c} \varepsilon_{11},\;t<20,t>21,\;\\ \varepsilon_{12},\;20\leq t\leq 21,\;\\ \; \end{array}\varepsilon_{2}=\{\begin{array}{c} \varepsilon_{21},\;t<20,t>21,\;\\ \varepsilon_{22},\;20\leq t\leq 21,\;\\ \; \end{array} & & \end{array}$$ (8)

where $\varepsilon_{12}>\varepsilon_{11}$ and $\varepsilon_{22}>\varepsilon_{21}$.

To restore the values of the extended parameter vector **q**, which now includes additional parameters $\varepsilon_{11}$, $\varepsilon_{12}$, $\varepsilon_{21}$ and $\varepsilon_{22}$, we apply the inverse problem approach to the YVM data. The obtained values are shown in S1 Table in S2 Appendix and the corresponding solution of the full model (1–5, 8) is shown in Fig 3 by the red curve. We readily observe that the model with policing has more complicated properties and its solution is in better agreement with data than the baseline one. Firstly, we note that the full model describes better the fast decay in the protesters number after week 32, in good agreement with the data. Secondly (and perhaps more importantly), in contrast to the baseline model, the number of street protesters described by the model with policing is a non-monotonous function of time, hence accounting for the local minimum in weeks 5-8. We recall here that this trough and the subsequent rebond have been regarded as the ‘calendar effect’: as weeks 5 and 6 in the 2019 series of YVM street protests fell into the Christmas & New Year break, it is believed that the lower participation might have resulted from family bonds and similar constraints, so that the numbers returned to ‘normal’ once the public holidays were over. However, here we have shown that there is an alternative explanation to that: the lower participation could as well be a result of harsh police actions in the preceding week 4 of the protests.

We further mention here that the nonmonotone behaviour of the solution can be explained mathematically by linking it to the properties of the corresponding dynamical system. It was shown in our earlier work (see Ref. [32]) that, in a certain parameter range the phase space of the system (1–4) contains a saddle point. For the initial conditions as in Eqs. (7), the system’s trajectory first approaches the saddle along the attracting manifold before turning away, cf. the shallow minimum of the red curve in week 3 in Fig 3). The change in parameters $\varepsilon_{1}$ and $\varepsilon_{2}$ in week 4 makes a perturbation that changes the direction of the phase flow, pushing it again towards the saddle (see the fast decay of the red curve approximately between days 20–35). Eventually, however, the trajectory is once again turned away along the repelling manifold, hence resulting in a nonmonotone change in the dynamical variables (see also Ref. [44] for examples of similar dynamics).

**Fig 4:**
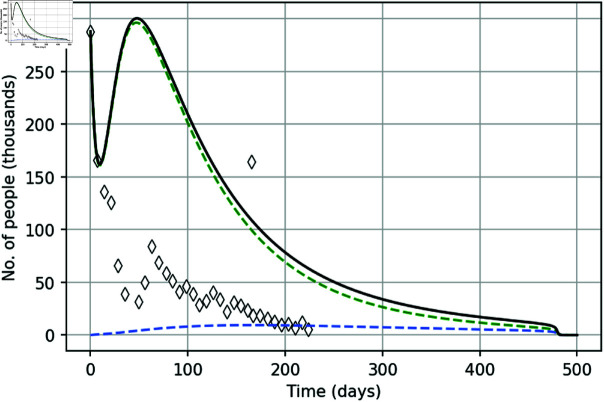
The total number of protesters in the absence of policing. The total number of protesters *I* ( *t* ) + *C* ( *t* )  (solid black curve) in the absence of policing, i.e. obtained for $\varepsilon_{11}=\varepsilon_{12}=\varepsilon_{21}=\varepsilon_{22}=0$, other parameter are the same as in Fig 3. The green and blue dashed curves show the numbers of novice and experienced protesters, respectively.

In order to further reveal the effect of policing on the course of protests, we now show the solution of the model (see Fig 4) in a hypothetical case with no police actions, i.e., setting $\varepsilon_{11}=\varepsilon_{12}=\varepsilon_{21}=\varepsilon_{22}=0$ and keeping other parameters the same as in Fig 3 (i.e., as given by parameter set **q**). It is readily seen that, without policing, the simulated street protests become much more intense. A decrease in the number of participants during the first three weeks is followed by an increase to a value that exceeds the one on the first day of the protests. Also, the overall duration of the protests increases more than twice.

#### Street protests control.

**Fig 5:**
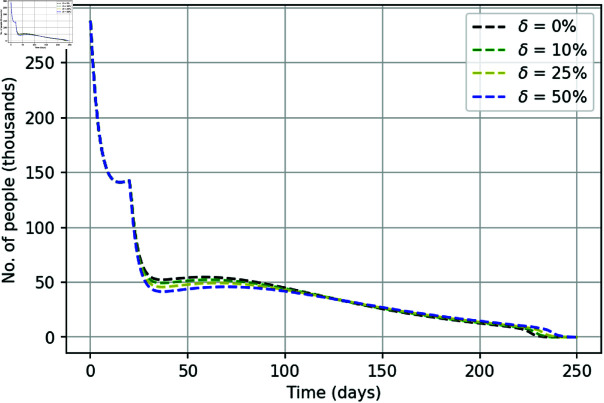
Number of street protesters  ( *C* + *I* )  over time obtained when $\varepsilon_{12}$ and $\varepsilon_{22}$ are raised by a certain percentage *δ*, i.e., $\varepsilon_{12}\to [1+(\delta ∕100)]\varepsilon_{12}$ and $\varepsilon_{22}\to [1+(\delta ∕100)]\varepsilon_{22}$.

Based on the above results, we can confidently assume that policing in general, and in particular the ‘active phase’ on day 21, is a factor that can be used to control or manage the course of the street protests. To make a further insight into the effect of the active phase, we now consider what could have been the course of the YVM movement in a hypothetical case of stronger police actions, hence using parameters $\varepsilon_{12}$ and $\varepsilon_{22}$ as controlling parameters. Given the definition of these parameters, an increase in their value is readily interpreted as either a corresponding increase in the number of police force involved in the protests policing or an increase of the number of detainees per policeman. Our simulation results shown in Fig 5 (for more details, see also S2 Fig in S4 Appendix). An increase in the intensity of single police actions on the 21st day leads to a slight decrease in the number of daily protesters.

Additionally, our model also helps to reveal the different roles that novice and experienced protesters play in the movement. Our simulation results (see S3 Fig. in S4 Appendix) show that, in case an increase in the efficiency of policing applies only to novice protesters but not to experienced ones (e.g., assuming that experienced protesters are much more difficult to catch), it has little or no effect on the course of street protests.

**Fig 6:**
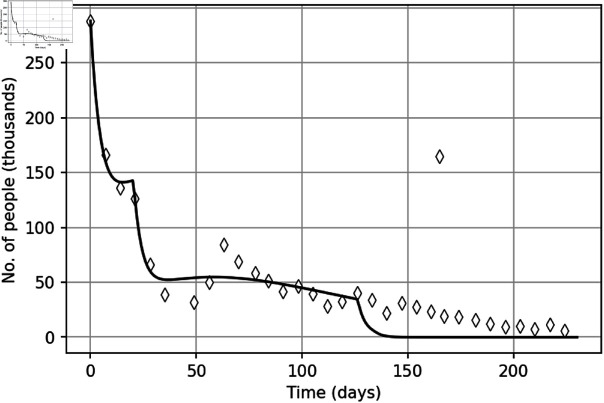
The course of the street protests obtained for parameter set q in a hypothetical case where another (additional) active phase of policing (described by a corresponding increase in $\varepsilon_{1}(t)$ and $\varepsilon_{2}(t)$) is applied on day 126.

In order to make a more detailed insight into the effect of policing, we now assume that another active phase of protests management took place on day 126. Correspondingly, in our model (1–5) we consider parameters $\varepsilon_{1}$ and $\varepsilon_{2}$ changing their values not only on day 21 but also on day 126, to the same values $\varepsilon_{12}$ and $\varepsilon_{22}$ as on day 21 (see S5 Appendix for details). Introduction of this additional active phase has a drastic effect on the course of protests, see Fig 6. The number of protesters drops sharply after day 126, leading to protests termination after just a few following weeks, much sooner than it happens without such additional control.

#### Protests prediction and further control.

**Fig 7:**
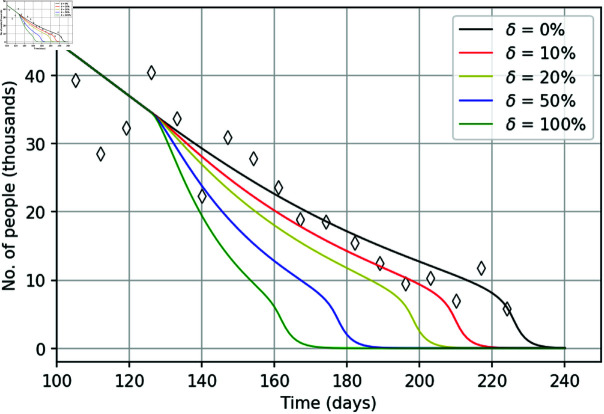
Number of street protesters  ( *C* + *I* )  over time during the second half of the YVM movement (*t* > 126) obtained in simulations using parameter set q for an increase in parameters $\varepsilon_{1}$ and $\varepsilon_{2}$ after day 126 by *δ* (in percent), i.e. for $\varepsilon_{1}\to [1+(\delta ∕100)]\varepsilon_{1}$ and $\varepsilon_{2}\to [1+(\delta ∕100)]\varepsilon_{2}$.

To make a further insight into the effect of policing, we consider another hypothetical scenario where the police shifts to a more efficient mode of protests control at day 126 and applies this mode until the end of protests. Correspondingly, this active phase now extends over entire later time *t* ≤ 126 (see S6 Appendix for more details). We additionally consider that, due to its long duration, this active phase is described somewhat differently compared to the one used on day 21 (as in Eqs. (8)), i.e., by increasing the basic detention rates $\varepsilon_{11}$ and $\varepsilon_{21}$ by a certain percentage, say *δ*. Result are shown in Fig 7 and they further confirm that policing is a crucial controlling factor. An increase in the efficiency of police actions by *δ* = 100% (green curve) reduces the duration of the second half of the protest movement more than twofold, making it approximately 50 days (after day 126) instead of 100 days given by the data or 140 days in the baseline case with no increase (*δ* = 0, black curve).

**Fig 8:**
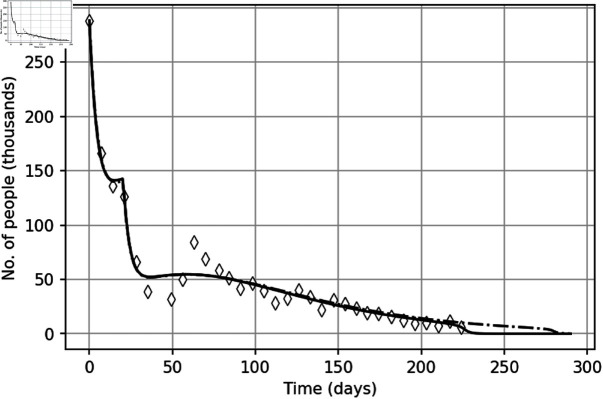
Solutions of the system –5, 8) with initial conditions (7) obtained for parameter set q (solid curve) and for parameter set q_1_ (dashed curve) restored from a ‘training set’ (truncated data). Parameter values are given in S1 Table.

In order also to check the predictive power of our model, we now restore the value of model parameters using only a subset of data (‘training set’), namely protests participants numbers between days 1-126, and see how well the corresponding solution describes the course of protests for *t* > 126. Namely, we first solve the inverse problem using data from 1 to 126 days to find a set of parameters **q**_1_ (see S1 Table in S1 Appendix) and then solve the direct problem using the reconstructed parameter set **q**_1_ over the entire time interval and compare it with real data. It appears that the corresponding solution of the model describes the rest of the data quite well, see Fig 8. It does not describe the apparent fast decay at the tail; however, we recall here that sporadic protests with a much smaller participant number actually continued after week 32, although reliable data are not available.

## Sensitivity

In this section, we estimate the identifiability of unknown parameters using sensitivity analysis. Consider the parameter vector as **q**=(*β*_1_,*β*_2_,*χ*,*n*,*C*_0_,*δ*_1_,*δ*_2_,*ε*_1_,*ε*_2_). In order to analyse the sensitivity of these nine parameters of our mathematical model (1–5, 8), we follow the approach previously used in Ref. [32] for the ‘baseline’ model that does not account for detained participants (*D* ( *t* ) ≡ 0).

Sensitivity analysis methods are based on the construction of a sensitivity matrix [45]. Let $t_{1}\leq t_{2}\leq \ldots \leq t_{N}$ be fixed time points at which a certain quantity *f* is ‘measured’ (in our case, the number of protests participants at a given date). Then the coefficients of the sensitivity matrix for the parameter vector **q**=(*q*_1_,…,*q_J_*) are calculated as follows:


$$\begin{array}{ccc} s_{ij}=q_{j}\frac{\partial f(t_{i},q)}{\partial q_{j}},\;j=1,\ldots ,J, & & \end{array}$$ (9)

(in our case *J* = 9) where *i* = 1 , … , *N* is the number of available data points (measurements), $q_{j}$ is the *j*th component of the parameter vector, $I(t_{i},q)\;+\;C(t_{i},q)=f(t_{i},q)$ are the inverse problem data. The quality of the parameter values estimation therefore depends on the number and location of available data points.

The sensitivity coefficients describe the system output’s response to changes in a single parameter. The system’s behaviour can be influenced by a parameter that is highly sensitive to a change in it. In contrast, a parameter that exhibits no impact on the outputs is a potential candidate for an unidentifiable parameter.Linearly dependent columns of the sensitivity matrix imply that a change in the system outputs due to a change in one parameter, say $q_{j}$, can be compensated by changing some or all of the dependent parameters $q_{k}$, with *k* ≠ *j*. The orthogonal method are based on this idea.

Let us consider the semi-relative sensitivity function for the solution vector **x**=(*S*,*I*,*C*,*R*,*D*) to calculate the coefficients of the sensitivity matrix:


$$\begin{array}{ccc} s_{q_{j}}(t)=q_{j}^{*}\frac{\partial x(t)}{\partial q_{j}},\;j=1,\ldots ,9. & & \end{array}$$ (10)

**Fig 9:**
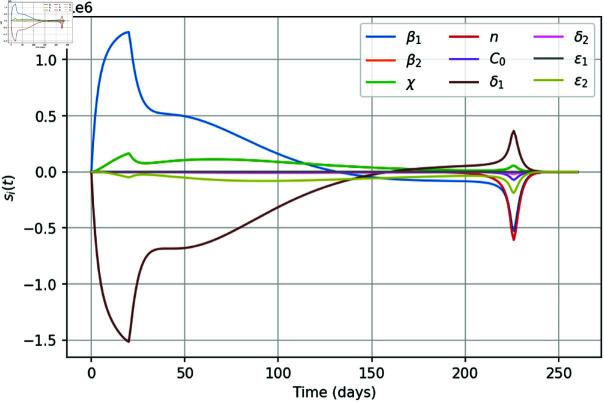
Sensitivity functions for all parameters of the system (1–5, 8) obtained for the ‘measured’ variable  ( *I* + *C* ) , with simulations performed for parameter set q (cf. S1 Table in S2 Appendix).

Figure 9 shows the components of the sensitivity matrix as functions of time obtained for parameter set **q** (cf. S1 Table in S2 Appendix).

We readily observe that the quality of parameter value estimation for $\delta_{2}$, $C_{0}$, *n* and $\varepsilon_{1}$ shows only a weak dependence on the amount of data on the number of protests participants  ( *I* + *C* ) , while the values of the remaining parameters exhibit a significant response to the amount of data. The parameters $\beta_{1}$ and $\delta_{1}$ are very sensitive in the first weeks of the social movement, which means that it is important to have a large amount of data at this moment in time to accurately determine them. For all parameters, there is an increase in the reaction of the system’s output in the final phase of the social movement, which indicates the difficulty of accurately predicting dynamics without having data in recent days. We also notice that, interestingly, the graphs of sensitivity functions for parameters $\beta_{2}$ and *χ* coincide.

In order to investigate the parameters’ identifiability, we apply the orthogonal method [46]. The method requires a certain parameter set as a starting point, for which we will use set **q** (see S1 Table in S2 Appendix). The basic idea of this approach is to examine the (nearly) linear dependencies of columns of the sensitivity matrix (9). To define a set of identifiable parameters, it is therefore possible for both the sensitivity of the system response to parameter values and the dependency between parameters regarding their effects on the system responses.

In practice, the orthogonal method consists of the following algorithm [46,47]:

1. Define an array of indexes of identifiable parameters *I* = ∅  and an array of unidentifiable parameters *U* = { 1 , … , *J* } .
2. Select the column *k* of the matrix *S* with the largest sum of squares of elements, add it to the matrix *E*, remove it from *S*. We add the index *k* to *I*, remove it from *U*.
3. If *U* = ∅ , then stop, else go to step 4.
4. Find orthogonal projections of vectors from *S* to the subspace of *E* and construct a matrix of perpendiculars $S^{⊥}$ to the matrix *S* consisting of *h* remaining columns:
  $$S_{p}^{⊥}=S_{p}-S_{p}^{proj},\;S_{p}^{proj}=\overset{J-h}{\underset{i=1}{\sum }}\frac{\left(S_{p},E_{i}\right)}{\left(E_{i},E_{i}\right)}E_{i}.$$
  Here $E=(E_{1},\ldots ,E_{J-h})$, *p* = 1 , … , *h*.
5. Select the column *k* of the matrix $S^{⊥}$ with the largest sum of squares of elements, add the index *k* to *I* and remove from*U*. Correspondingly, remove the corresponding column from *S* and add it to the matrix *E*. Go to step 3.

**Fig 10:**
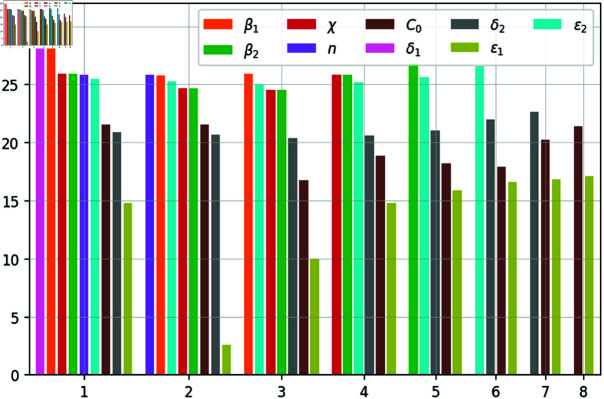
The norm of the perpendiculars (in logarithmic scale) calculated at each iteration of the orthogonal method. The horizontal axis shows the succession of algorithm’s iterations (eight altogether).

The results of the parameter identifiability analysis for our model (1–5, 8) at various iterations of the orthogonal method are presented in Fig 10. A sequence of parameters (from the most to the least identifiable) is obtained: $\delta_{1}$,  − *∞*, $\beta_{1}$, *∞*, $\beta_{2}$, $\varepsilon_{2}$, $\delta_{2}$, $C_{0}$, $\varepsilon_{1}$. Note the dependence between the parameters $\delta_{1}\text{and}\varepsilon_{1}$. It means that, while the effect of other parameter on the model’s predictions is unique, the influence of $\varepsilon_{1}$ on the *∞* can be partially compensated by changes in parameter $\delta_{1}$.

## Discussion and conclusions

Social protests, in particular in the form of street protests, are a ubiquitous feature of the modern society, often being an important driver of change in the society and social system. Street protests, especially when occurring on a massive scale, can also bring a considerable disruption to normal course of life and cause significant loss and damage. Understanding factors that can affect intensity and duration of street protests is therefore a problem of high practical importance. Mathematical modelling has been increasingly recognized as powerful and convenient research tool to identify such factors and to evaluate their relative importance [18,22,30,31,33]. Once relevant factors are revealed and their effect understood, it opens a possibility of protest management [31,48], e.g., through advising relevant authorities in order to help them to make informed decision aiming at minimizing the disruption and ensuring public safety.

While there is a vast social science literature addressing this problem qualitatively, quantitative approaches to protests description and/or modelling are still at their early stage. There is relatively little literature concerned with mathematical modelling of street protests, demonstrations, riots, crime, or social unrest more generally. The choice of modelling framework depends on the focus and objectives of a specific study, in particular whether the spatial aspects of corresponding processes and dynamics are explicitly considered. Examples of spatially explicit modelling approaches are given by geostatistics [9,11], agent-based simulations [18,21] and diffusion-reaction equations where the latter can be used in continuous space [49,50] as well as on networks [51]. However, when street protests occur in a relatively compact spatial area (e.g., the YVM weekly events mostly happened in Paris, with a much small number of participants in other cities), we argue that spatially explicit approaches are not necessary and indeed conceptual non-spatial or spatially-implicit ‘mean-field’ modelling frameworks have been developed [15,29,52–56].

In this paper, based on our previous work [32], we presented a non-spatial compartment-type mathematical model of the protests dynamics that describes protests as an ‘epidemic’ of a certain behaviour [29] and hence use ideas from disease modelling. An essentially new feature of our model is that it accounts for the effect of policing. Using data on the Yellow Vest Movement in France, we showed that, not only the model describes the course of street protests quite well and have a certain predictive power, but it also suggests possible ways of protests management and control. In particular, we have shown that an increase in the efficiency of policing by just 10–20% can sometimes decrease the protests activity more than twice.

We mention here that, although the idea of using the police to control and manage social unrest as such is by no means new [14,18,22,31], it also instigated certain doubts about its efficiency (cf. [57]) and the issue as a whole remains controversial. In particular, a recent study by Adam-Troian et al. [58] found that police actions may in fact radicalize some of the affected protesters, hence potentially increasing their determination to participate in further street protests. (We, however, note here that, as is admited by the authors of Ref. [58], their sample might have not been large enough to generalize their findings across the whole cohort of the YVM participants and likely consisted of already highly politicized individuals.) Our study contributes to this discussion by showing that measured, proportionate police actions can be efficient protests management strategy not only in a short term, e.g. for a single protest, but also in a longer term, i.e. changing the course of the whole series of events. We emphasize here that our model does not as such assume that a higher level of repression should necessarily reduce protests activity. Although this effect has indeed been seen in our simulation results, it appears to be an intrinsic, emerging property of the model but not our assumption per se.

With regard to the capacity of our model to describe different protests patterns, we recall here that our choice of the initial conditions, cf.(), was made consistent with the data on the YVM where the first event was attended by the largest number of people. While this pattern is not unique (street protests in Khabarovsk in 2019 showed a very similar behaviour, see Ref. [32]), some other protests or riots (e.g., French riots in 2005, see Ref. [30]) showed a different pattern, i.e., starting with a small number at the beginning but then rising relatively fast to a high peak. This different pattern can be described by our model as well. In fact, already our baseline model given by –4) with *#*3 can exhibit a single-peak behaviour consistent with protests data (apparently for a different choice of the initial conditions); see Ref. [56] for details.

Note that our modelling approach is based on certain simplifying assumptions about human behaviour. In particular, our sorting of the whole broad variety of human behavioural responses and patterns into just two archetypes, i.e., ‘novice’ and ‘experienced’, is at best only a caricature of reality. This approach could be refined by adding other behavioural types or by including different grades of experience. For example, there is anecdotal evidence of the existence of professional trouble-makers (not necessarily the same as our ‘mature protesters’) who would usually join the otherwise peaceful protest with the purpose to bring violence and disorder. Explicit inclusion of this and similar groups could be an interesting extension of the model, especially in the case where loss and damage is explicitly included into consideration (cf. Refs. [50,56]). As a drawback of this approach, however, we mention that a more complicated and/or more detailed model would inevitably contain additional parameters whose value may be difficult to estimate and that may increase the uncertainty of the model predictions.

Similarly, for the purposes of this paper, we do not distinguish between protests and riots. Although they may seem to be significantly different—indeed, while a protest can be entirely peaceful, a riot is endemically violent - it remains unclear whether this difference is fundamental or riots and peaceful protests are just two extremes in a continuous spectrum of all possible types where the level of violence may change gradually from very low (ultimately, being absent altogether) to very high. Incorporating these differences into the model lies beyond the scope of our present paper but should become a focus of future work.

Our study leaves a few open questions. Firstly, recall that, according to our definition, the susceptible group includes all general population of the country. Although arguably being a reasonable assumption, it is also a strong simplification. A more advanced model could for instance consider a division of the general population into two groups consisting of people who would, respectively, support or opposed the movement, with possible transitions between the groups. Or, alternatively, the total number of potential protesters could be made time-dependent, e.g. being considered as a variable determined by a certain stochastic process. These generalizations lie beyond the scope of the present paper but should become a focus of future research.

Secondly, in our model we have assumed that the interactions between different groups of active or potential protests participants (i.e.  − 0 . 79 > *r* > 0 . 09, *r* > 0 . 09, *r* < − 0 . 79 and  − 0 . 15 > *r* > 0 . 15) that may affect their opinion to join or withdraw from the protests take place due to direct ‘face to face’ communication. Apparently, this mode of communication, although undoubtfully important, entirely ignores an alternative ‘indirect’ communication through social media. Meanwhile, this is known to be important [23,59]. Moreover, the effect of traditional mass media like the TV and newspapers is not taken into effect either. The latter however can also be an important factor, as it mounts a certain information pressure that can act both in favour of the protests as well as against it. How these factors can be included into the model and how they can change the model properties (e.g. its predictive power) remain unclear.

One generic fundamental problem with street protests modelling is the reliability of the data on the number of protesters. In particular, in the case of YVM, data available from different sources can differ almost by an order of magnitude (see S7 Appendix). This is perhaps not surprising: apart from purely technical issues related to protesters counting, the number of protesters is a highly sensitive political issue and as such is susceptible to all sorts of speculations and manipulations [42,60]. In turn, it suggests that a more advanced modelling approach should account for uncertainty in the data. One possible way to incorporate the uncertainty is to add a random perturbation to the data, e.g. as ‘noise’ [32]. A more general approach could also use the ideas of fuzzy modelling [61]. Development of an efficient modelling approach incorporating the uncertainty should become a focus of future work.

## Supporting information

S1 Text Appendices S1–S7. (PDF)
