# Correction: Understanding street protests: From a mathematical model to protest management

**DOI:** 10.1371/journal.pone.0345122

**Published:** 2026-03-16

**Authors:** Sergei Petrovskii, Maxim Shishlenin, Anton Glukhov

The Funding statement for this article is incorrect. The correct Funding statement is as follows: M. Shishlenin and A. Glukhov acknowledge the support through the state assignment of IM SB RAS (Theme No. FWNF-2024–0001). S. Petrovskii acknowledges support by the RUDN University through Strategic Academic Leadership Program.
