# Supplementary material for: Understanding street protests: from a mathematical model to protest management
Source: PLoS One. 2025 Apr 10;20(4):e0319837. doi: 10.1371/journal.pone.0319837 (PMC11984982; doi:10.1371/journal.pone.0319837)
Supplement: S1 Text — (PDF) [file pone.0319837.s001.pdf]

Understanding street protests: from a mathematical model to protest management

Sergei Petrovskii, Maxim Shishlenin, Anton Glukhov

Supporting information

S1 Appendix. Fitting model to data: inverse problem method.

To determine the parameter values in the model given by Eqs. (1-5) in the main text, we consider it as an inverse problem (e.g. see Refs. (1; 2)). Since no data are available on the relative numbers of novice and experienced protesters, to compare with the available data on protesters numbers, we use the sum of $I(t) + C(t)$.

The general inverse problem approach adapted to our system looks as follows. Let the following data set is available (where K, N, I are the numbers of the corresponding data points/days):

$$I(t_k) + C(t_k) = g_k, \qquad k = 1, \dots, K, \tag{1}$$

$$D(t_p) = d_p, \qquad p = 1, \dots, P. \tag{2}$$

$$\hat{D}(t_n) = D(t_n) - D(t_{n-1}) = \hat{d}_n, \qquad n = 1, \dots, N, \tag{3}$$

where Eq. (3) is used in case the number of arrests is known on the specific date t_n; otherwise Eq. (2) is used (see below).

The inverse problem approach aims to find the vector $\mathbf{q} = (q_1, \dots, q_M)$, where M is the number of unknown parameters in the model (Eqs. (1-5) in tne main text) that provides the 'best fit' (in some sense, see below) of the model solution to the observation data, cf. Eqs. (1-3). Using *a priori* information about the desired solution to the inverse problem can greatly reduce the requirements for computing resources, see Ref. (3).

In general mathematical terms, the inverse problem defined by Eqs. (1-5) in the main text combined with Eqs. (1-3) above can be formulated as follows:

$$\mathcal{A}(\mathbf{q}) = f, \qquad \mathcal{A} : \mathbf{R}^M \to \mathbf{R}^{K+N+P} \quad \Rightarrow \quad \mathbf{q} = \mathcal{A}^{-1}(f), \tag{4}$$

where $\mathcal{A}$ is the corresponding operator and f is the set of all available statistics.

Thus, the inverse problem (4) is reduced to a more standard problem of minimizing the

following functional:

$$F(\mathbf{q}) = \sum_{k=1}^{K} [I(t_k;\mathbf{q}) + C(t_k;\mathbf{q}) - g_k]^2 + \sum_{n=1}^{N} \left[\hat{D}(t_n;\mathbf{q}) - \hat{d}_n\right]^2 + \\ + \sum_{p=1}^{P} [D(t_p;\mathbf{q}) - d_p]^2 \longrightarrow \min_{\mathbf{q}}. \quad (5)$$

Here $I(t;\mathbf{q})$, $C(t;\mathbf{q})$, $D(t;\mathbf{q})$ are the solution of the direct problem for fixed $\mathbf{q}$. To find the global minimum of the functional (5), a global mathematical optimization method (differential evolution, see Ref. (4)) is used.

Now we recall that the available data gives the number of arrests but not the number of detainees. In case the number of protesters arrested on the date t_i is known - say, A_i - then it is readily seen that $\hat{d}_n = A_n$ (see Eq. 3). In case only a cumulative number of arrests by a given date (t_p) is known, in order to obtain an estimate for d_p, we use the following approach. We have previously assumed that the arrested people are eventually released with a certain release rate ε_3 (cf. Fig. 1 in the main text). It means that the corresponding number of detainees decreases with time exponentially, so that by the date $t_p \geq t_i$ the number of people remaining in custody is $A_i \exp[-\varepsilon_3(t_p - t_i)]$. Therefore, the total number of detainees at date t_p estimated from the data on arrests number is

$$d_p \equiv d(t_p) = \sum_{i=1}^{i=p} A_i \exp[-\varepsilon_3(t_p - t_i)]. \quad (6)$$

Direct application of the above estimate could only be possible if the number of arrests were available at each preceding protest date, i.e. $A_1, A_2, \ldots, A_p$. Instead, however, only the cumulative number is available, i.e. $\bar{A} = A_1 + A_2 + \ldots + A_p$. Therefore, in order to make a comparison between the model and the data possible, we have to make an additional assumption about the pattern of arrests over time. Namely, here we assume that the weekly number of arrests is constant (say a_1), so that $A_1 = A_2 = \ldots = A_p = a_1$. Correspondingly, Eq. (6) becomes

$$d_p = a_1 \sum_{i=1}^{i=p} \exp[-\varepsilon_3(t_p - t_i)].$$

The weekly number of arrests can be estimated from the total as $a_1 = \bar{A}/n_e$ where n_e is the number of protests in the series (essentially, the number of weeks, as the YVM protests were normally held on Saturdays). Since the data indicates that the average weekly number of arrests tended to decrease with time (see Section "Data" in the main text), we consider the first half ($0 < t \leq 126$) and the second half ($126 < t \leq 259$) of the protests separately, so that a_1 and n_e take different values for the first and second halves accordingly.

Once the parameter values are obtained, to solve direct problem described by Eqs. (1-5) in the main text, the Runge-Kutta-Merson method of 4th order is applied.

S2 Appendix. Model parameters.

Parameters used in our model (see Eqs. (1-5) in the main text) obtained using the inverse problem method (cf. Appendix S1) are given in Table 1 below.

Table 1: **Values of the model parameters obtained from the YVM data using the inverse problem approach.**

	Parameter set $\mathbf{q}_*$	Parameter set $\mathbf{q}$	Parameter set $\mathbf{q}_1$
β_1	$0.1941 \cdot 10^{-10}$	$0.1958 \cdot 10^{-7}$	$0.2093 \cdot 10^{-7}$
β_2	$0.1045 \cdot 10^{-6}$	$0.6583 \cdot 10^{-6}$	$2.0452 \cdot 10^{-6}$
χ	$0.1632 \cdot 10^{-3}$	$0.4051 \cdot 10^{-3}$	$0.1226 \cdot 10^{-3}$
n	1.4993	7.2991	9.6897
C_0	27975	5323	3157
δ_1	0.0684	1.2017	1.2691
δ_2	$0.2167 \cdot 10^{-3}$	$0.4281 \cdot 10^{-2}$	$0.9623 \cdot 10^{-2}$
ε_{11}	-	$0.6421 \cdot 10^{-3}$	$0.8481 \cdot 10^{-3}$
ε_{21}	-	0.0421	0.0306
ε_{12}	-	$0.6309 \cdot 10^{-2}$	$0.4092 \cdot 10^{-2}$
ε_{22}	-	2.032	1.8743
F_2	$4712 \cdot 10^6$	$2305 \cdot 10^6$	$2360 \cdot 10^6$

S3 Appendix. Policing without active phases.

Consider the situation where the removal of active protests participants by police remained unchanged throughout the whole duration of protest movement. To do so, we simulate the course of the protests using parameter set $\mathbf{q}$, but replace the active phase of external influence with a weak one, i.e., $\varepsilon_{12} \equiv \varepsilon_{11}$ and $\varepsilon_{22} \equiv \varepsilon_{21}$. Result are shown in Fig. 1). It is readily seen that, in the absence of active police control, protests become more massive in the first three months, in particular almost entirely missing the drop in numbers that happened between weeks 3-8. However, the overall duration of the protests becomes several weeks shorter.

S4 Appendix. Differential effect of policing on novice and mature protesters

Figure 2 shows the course of street protest in the case where the efficiency of police control during the weak and active phases (as is quantified by parameters ε_{11}, ε_{21}, ε_{12}, ε_{22}, see (**??**)) was increased by δ (in %), i.e. changing their respective values as $\varepsilon_{11} \to [1 + (\delta/100)]\varepsilon_{11}$, $\varepsilon_{21} \to [1+(\delta/100)]\varepsilon_{21}$, $\varepsilon_{12} \to [1+(\delta/100)]\varepsilon_{12}$, $\varepsilon_{22} \to [1+(\delta/100)]\varepsilon_{22}$. Obviously, such control

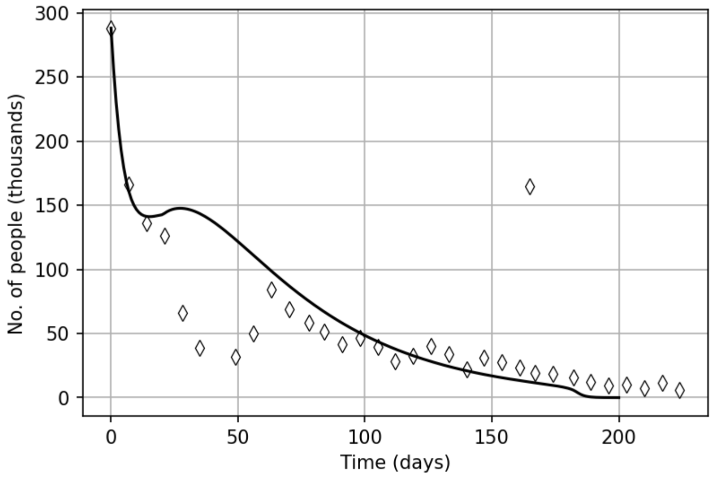

Figure 1: The dynamics of the protest movement corresponding to the parameter set **q**, with the active phase replaced by the weak phase, i.e. $\varepsilon_{12} \equiv \varepsilon_{11}, \varepsilon_{22} \equiv \varepsilon_{21}$.

has a significant effect on the course of the protests both in terms of participants numbers and overall protests duration.

For comparison, Fig. 3 shows the course of street protest in the case where the increase in police efficiency only applies to novice protesters, but not to experienced ones (i.e. keeping parameters ε_{21} and ε_{22} unchanged). Apparently, except for an increase in the number of detained participant, control of this type has practically no effect on the daily numbers of

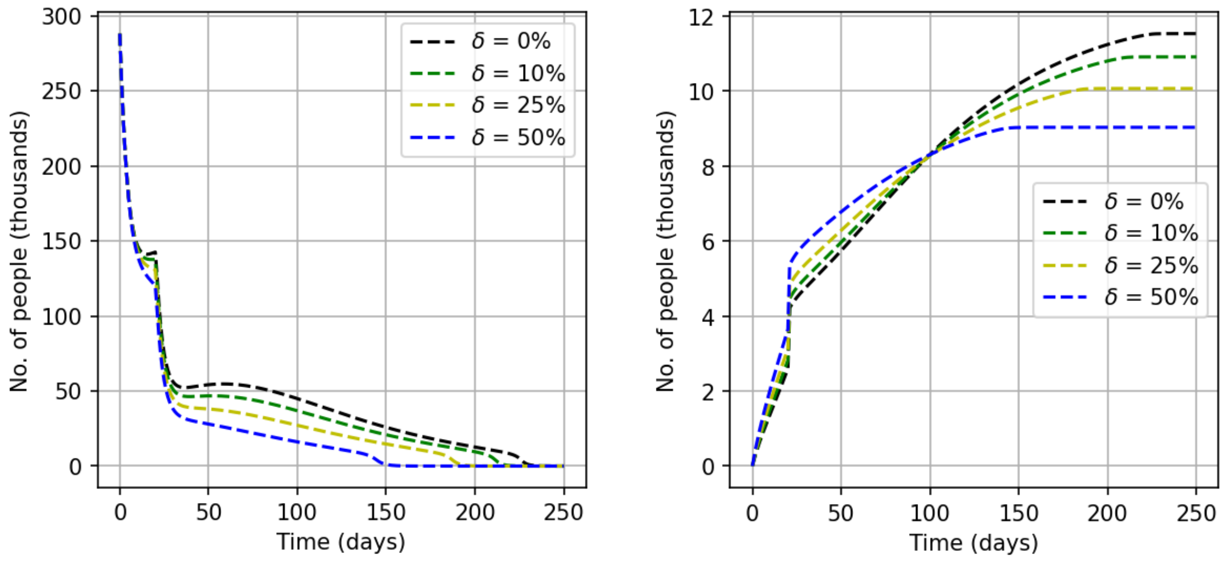

Figure 2: The number of active street protesters $(C + I)$ (left panel) and the number of detained protesters $D(t)$ (right panel) obtained for an increase in the efficiency of the weak and active phases of policing (quantified by parameters ε_{11}, ε_{21}, ε_{12}, ε_{22}) by δ (in percent).

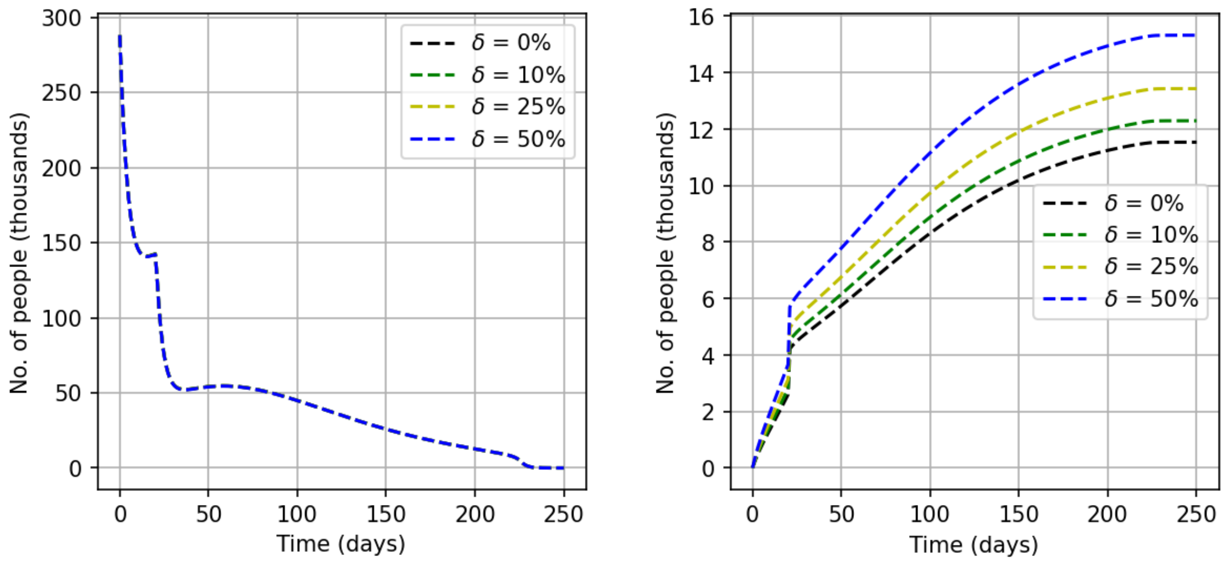

Figure 3: The number of active street protesters $C + I$ (left panel) and the number of detained protesters $D(t)$ (right panel) in the case where during the weak and active phases of police control only parameters ε_{11} and ε_{12} increases by $\delta\%$, i.e. $\varepsilon_{11} \to [1 + (\delta/100)]\varepsilon_{11}$ and $\varepsilon_{12} \to [1 + (\delta/100)]\varepsilon_{12}$. Note that the five curves in panel (a) lie very close to each other, giving an impression of a single curve.

active street protesters and the overall duration of the movement.

S5 Appendix. Additional active policing on day 126

To account for the extra active phase, parameters ε_1 and ε_2 are adapted as follows:

$$\varepsilon_1 = \begin{cases} \varepsilon_{11}, & t < 20, \quad 21 < t < 126, \quad t > 127, \\ \varepsilon_{12}, & 20 \leq t \leq 21 \quad \text{and} \quad 126 \leq t \leq 127, \end{cases}$$

$$\varepsilon_2 = \begin{cases} \varepsilon_{21}, & t < 20, \quad 21 < t < 126, \quad t > 127, \\ \varepsilon_{22}, & 20 \leq t \leq 21 \quad \text{and} \quad 126 \leq t \leq 127, \end{cases}$$

where $\varepsilon_{11}, \varepsilon_{12}, \varepsilon_{21}$ and ε_{22} are additional parameters that can be obtained from analysing available data.

S6 Appendix. Switch to active policing after day 126

To account for more active policing in the second half of the protests (after day 126), pa-

rameters ε_1 and ε_2 are adapted as follows:

$$\varepsilon_1 = \begin{cases} \varepsilon_{11}, & t < 20, \quad 21 < t < 126, \\ \varepsilon_{12}, & 20 \leq t \leq 21, \\ [1 + (\delta/100)]\varepsilon_{11}, & t \geq 126, \end{cases}$$

$$\varepsilon_2 = \begin{cases} \varepsilon_{21}, & t < 20, \quad 21 < t < 126, \\ \varepsilon_{22}, & 20 \leq t \leq 21, \\ [1 + (\delta/100)]\varepsilon_{21}, & t \geq 126, \end{cases}$$

where the increase $\delta > 0$ is given in %.

S7 Appendix. Alternative sources of data

One of the serious problems in the study of social protests is the lack of reliable data. In the case of Yellow Vest Movement, there are several official or 'semi-official' sources that provide significantly different data on the participants numbers: (i) The YVM page on social media which disseminated information about upcoming protests and provided statistics, cf. Ref. (5). (ii) The website of the French Interior Ministry, where brief reports on the actions were published (see Ref. (6)). (iii) Wikipedia collected the data obtained from a few different sources. An example is shown in Fig. 4 where the left and right panels correspond to Ref. (7) and Ref. (8), respectively.

An article in Le Monde discusses possible reasons why the data are apparently so different (9). One of the possibilities that was suggested was that at least sometimes number of participants was counted differently for political reasons, which led to a biased representation of French public opinion, see Ref. (10).

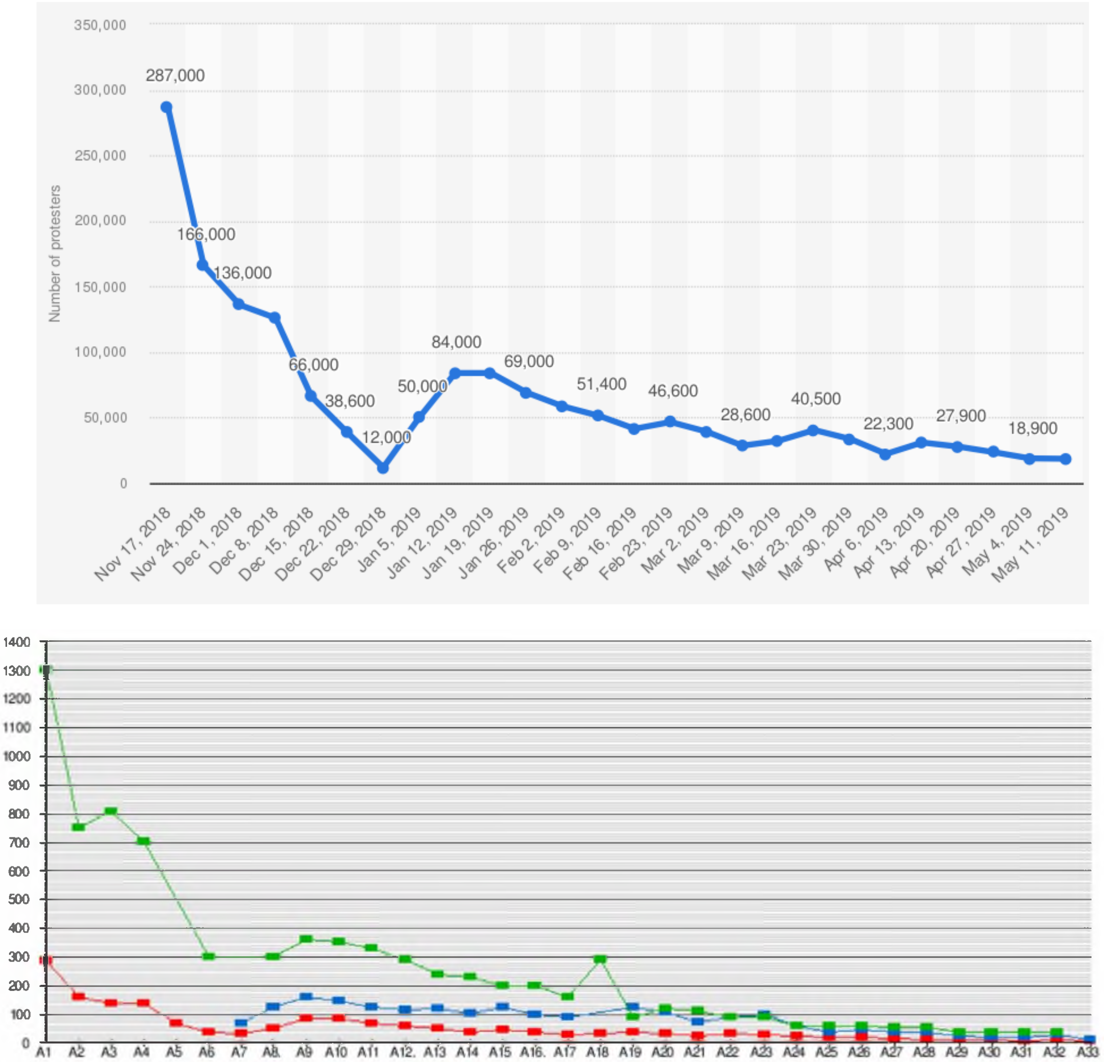

Figure 4: Number of protesters during the YVM events in France in 2018–2019. (top) Data from Statista Research Department (7), March 11, 2024. (bottom) Data aggregated by Wikipedia (8) (version from August 21, 2021). Red - data from French Interior Ministry, blue - Facebook, green - France Police Asociation “Angry Policemen”.

References

[1] A. Tarantola, Inverse problem theory and methods for model parameter estimation, SIAM, 2005.

[2] S. I. Kabanikhin, Inverse and Ill-posed Problems Theory and Applications, de Gruyter, 2011.

[3] S. I. Kabanikhin, M. A. Shishlenin, Quasi-solution in inverse coefficient problems, Journal of Inverse and Ill-Posed Problems 16 (7) (2008) 705–713.

[4] R. Storn, K. Price, Differential evolution - a simple and efficient heuristic for global optimization over continuous spaces, Journal of Global Optimization 11 (4) (1997) 341–359.

[5] Facebook/lenombrejaune, https://www.facebook.com/lenombrejaune/ (2022).

[6] A. French Interior Ministry, https://www.interieur.gouv.fr/Publications (2022).

[7] A. Statista Research Department, https://www.statista.com/statistics/952150/number-of-demonstrators-yellow-vests-france/ (2024).

[8] Wikipedia, Mouvement des gilets jaunes, https://fr.wikipedia.org/w/index.php?title=Mouvement-des-Gilets-jaunes&oldid=185894441 (2021).

[9] P. W. Audureau, Le Monde, https://www.lemonde.fr/les-decodeurs/article/2019/01/21/le-difficile-comptage-des-rassemblements-de-gilets-jaunes_5412352_4355770.html, january 21, 2019 (2019).

[10] B. Monnery, F. Wolff, Is participatory democracy in line with social protest? evidence from the french yellow vests movement, Public Choice (197) (2023) 283–309.
